# *N*-(6-Chloro-1-methyl-1*H*-imidazo[4,5-*c*]pyridin-4-yl)benzene­sulfonamide

**DOI:** 10.1107/S1600536814005388

**Published:** 2014-03-15

**Authors:** Venkatesh B. Devaru, K. S. Katagi, O. Kotresh, H. K. Arunkashi, H. C. Devarajegowda

**Affiliations:** a P. G. Department of Physics, LVD College, Raichur 584 103, Karnataka, India; b Department of Chemistry, Karnatak Science College, Karnatak University, Dharwad, Karnataka 580 001, India; c Department of Physics, Moodlakatte Institute of Technology, Kundapura 576 217, Karnataka, India; d Department of Physics, Yuvaraja’s College (Constituent College), University of Mysore, Mysore 570 005, Karnataka, India

## Abstract

The asymmetric unit of the title compound, C_13_H_11_ClN_4_O_2_S, contains two mol­ecules (*A* and *B*), in which the dihedral angles between the 1*H*-imidazo[4,5-*c*]pyridine system and terminal phenyl ring are 80.83 (10) and 62.34 (1)°. In the crystal, *A*–*B* dimers are linked by pairs of N—H⋯N hydrogen bonds, which generate *R* ^2^_2_(10) loops. The dimers are linked by C—H⋯O and C—H⋯Cl inter­actions, generating a three-dimensional network. Aromatic π–π stacking inter­actions [shortest centroid–centroid distance = 3.5211 (12) Å] are also observed.

## Related literature   

For biological background, see: Kulkarni & Newman (2007 ▶). For a related structure, see: Kandri Rodi *et al.* (2013 ▶).
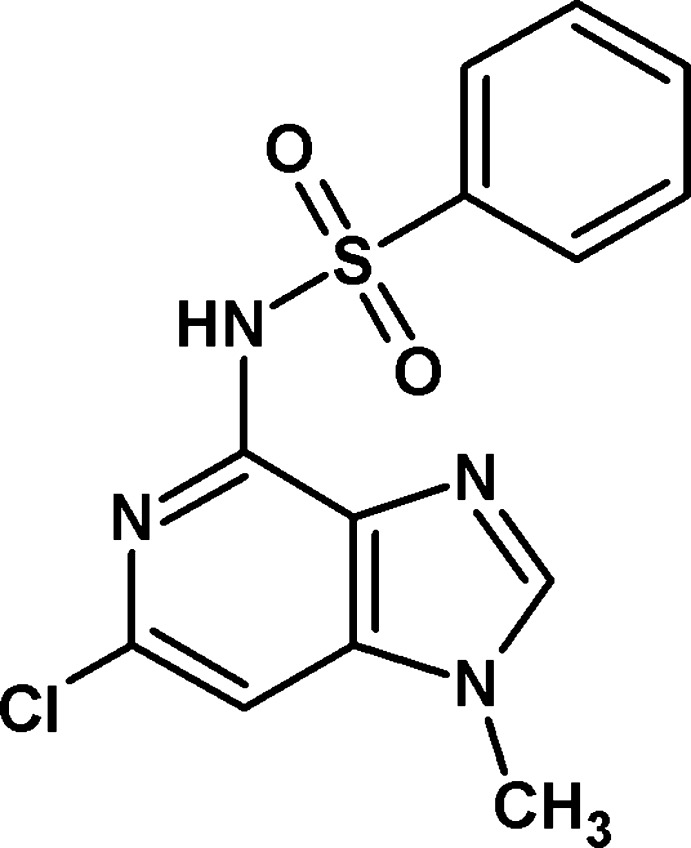


## Experimental   

### 

#### Crystal data  

C_13_H_11_ClN_4_O_2_S
*M* *_r_* = 322.77
Monoclinic, 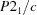

*a* = 13.1045 (4) Å
*b* = 14.7259 (5) Å
*c* = 14.6953 (6) Å
β = 95.127 (3)°
*V* = 2824.49 (17) Å^3^
*Z* = 8
Mo *K*α radiation
μ = 0.43 mm^−1^
*T* = 293 K
0.24 × 0.20 × 0.12 mm

#### Data collection  

Bruker SMART CCD diffractometer
Absorption correction: multi-scan (*SADABS*; Bruker, 2001 ▶) *T*_min_ = 0.770, *T*_max_ = 1.000
16038 measured reflections
4972 independent reflections
3759 reflections with *I* > 2σ(*I*)
*R*_int_ = 0.031

#### Refinement  

*R*[*F* ^2^ > 2σ(*F* ^2^)] = 0.034
*wR*(*F* ^2^) = 0.098
*S* = 1.04
4972 reflections
379 parameters
H-atom parameters constrained
Δρ_max_ = 0.30 e Å^−3^
Δρ_min_ = −0.31 e Å^−3^

### 

Data collection: *SMART* (Bruker, 2001 ▶); cell refinement: *SAINT* (Bruker, 2001 ▶); data reduction: *SAINT*; program(s) used to solve structure: *SHELXS97* (Sheldrick, 2008 ▶); program(s) used to refine structure: *SHELXL97* (Sheldrick, 2008 ▶); molecular graphics: *ORTEP-3 for Windows* (Farrugia, 2012 ▶); software used to prepare material for publication: *SHELXL97*.

## Supplementary Material

Crystal structure: contains datablock(s) I, global. DOI: 10.1107/S1600536814005388/hb7208sup1.cif

Structure factors: contains datablock(s) I. DOI: 10.1107/S1600536814005388/hb7208Isup2.hkl

Supporting information file. DOI: 10.1107/S1600536814005388/hb7208Isup3.cml

CCDC reference: 990803

Additional supporting information:  crystallographic information; 3D view; checkCIF report

## Figures and Tables

**Table 1:** Hydrogen-bond geometry (Å, °)

*D*—H⋯*A*	*D*—H	H⋯*A*	*D*⋯*A*	*D*—H⋯*A*
N7*A*—H7*A*⋯N8*B* ^i^	0.86	2.25	3.014 (2)	148
N7*B*—H7*B*⋯N8*A* ^ii^	0.86	2.25	3.092 (2)	165
C13*A*—H13*A*⋯Cl1*A* ^iii^	0.93	2.82	3.620 (3)	144
C20*A*—H20*A*⋯O4*A* ^iv^	0.93	2.47	3.243 (3)	141

Symmetry codes: (i) 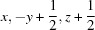; (ii) 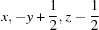; (iii) 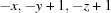; (iv) 
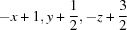
.

**Fig. 1.**
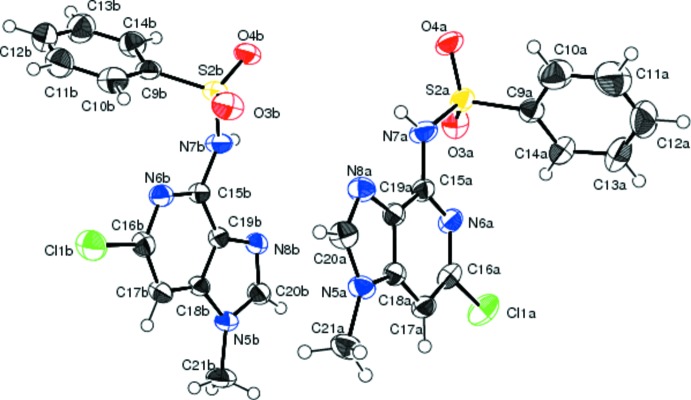
The molecular structure of the title compound. Displacement ellipsoids are drawn at the 50% probability level. Hydrogen bonds are shown as open dashed bonds.

## supplementary crystallographic information

### 1. Comment

Imidazo[4,5]pyridine derivatives have been reported as inhibitors of mitogen and stress-activated protein kinases and aurora kinases (Kulkarni & Newman, 2007). As part of our studies of this family of compounds, we now describe the synthesis and structure of the title compound. Its gometrical data are good agreement with those of a related structure (Kandri Rodi *et al.*, 2013)

The asymmetric unit contains two independent molecules A and B respectively are shown in Fig. 1. The 1*H*-imidazo [4,5-*c*]pyridine rings system are nearly planar, with a maximum deviations of 0.0284 (19) Å and 0.0061 (19) Å for the atoms C19*a* and C19*b*; the dihedral angles between the 1*H*-imidazo [4,5-*c*]pyridine (N5a/N6a/N8a/C15*a*–C20*a*) & (N5b/N6b/N8b/C15*b*–C20*a*) and the terminal benzene (C9a–C14*a*) & (C9b–C14*b*) rings are 80.83 (10)° and 62.34 (1)°.

In the crystal, A-B dimers are linked by pairs of N—H···N hydrogen bonds (Table 1), which generate R^2^_2_(10) loops. The dimers are lined by C—H···O and C—H···Cl interactions to generate a three-dimensional network. Aromatic π–π stacking interactions [shortest centroid–centroid distance = 3.5211 (12) Å] are also observed.

### 2. Experimental

A mixture of 2,4,6-trichloropyridene, methylamine in ethanol was heated and filtered to get pure product. To this sulfuric acid and fuming nitric acid was added, then it was stirred and cooled. A solution of iron powder and ammonium chloride in methanol/water was added and heated. Then triethylorthoformate in ethanol was added and continued the heating. Amination of reaction product was achieved by adding benzophenone imine, potassium carbonate, palladium complex, in dioxane, and then it was heated. The obtained product was dissolved in HCl, stirred, and concentrated *in vacuo* to give the product. A mixture of obtained product, sodium hydride, 6-chloro-1-methyl-1*H*-imidazol [4,5-*c*]pyridin-4-amine and carbonyl/sulfonyl chlorides in tetahydrofuran was stirred and concentrated *in vacuo* to give the expected products. After completion of each step of the reaction TLC was monitored. The compound is recrystallized by ethanol- chloroform mixture. Colourless needles of the title compound were grown from a mixed solution of ethanol/chloroform (v/v = 2/1) by slow evaporation at room temperature. Yield: 121 mg, 64.84%); mp: 336k; IR cm^-1^ (KBr) 3435, 1388; Anal. Calcd for C_13_H_11_ClN_4_O_2_S C, 45.82; H, 2.96; N, 16.44%; Found, C, 45.18; H, 2.92; N, 15.84%.

### 3. Refinement

All H atoms were positioned geometrically, with N—H = 0.86 Å, C—H = 0.93 Å for aromatic H and C—H = 0.96 Å for methyl H, and refined using a riding model with *U*_iso_(H) = 1.5*U*_eq_(C) for methyl H and *U*_iso_(H) = 1.2*U*_eq_(C) for all other H.

### Crystal data

**Table:**

C_13_H_11_ClN_4_O_2_S	*F*(000) = 1328
*M**_r_* = 322.77	*D*_x_ = 1.518 Mg m^−^^3^
Monoclinic, *P*2_1_/*c*	Melting point: 376 K
Hall symbol: -P 2ybc	Mo *K*α radiation, λ = 0.71073 Å
*a* = 13.1045 (4) Å	Cell parameters from 4972 reflections
*b* = 14.7259 (5) Å	θ = 2.8–25.0°
*c* = 14.6953 (6) Å	µ = 0.43 mm^−^^1^
β = 95.127 (3)°	*T* = 293 K
*V* = 2824.49 (17) Å^3^	Plate, colourless
*Z* = 8	0.24 × 0.20 × 0.12 mm

### Data collection

**Table:**

Bruker SMART CCD diffractometer	4972 independent reflections
Radiation source: fine-focus sealed tube	3759 reflections with *I* > 2σ(*I*)
Graphite monochromator	*R*_int_ = 0.031
ω and φ scans	θ_max_ = 25.0°, θ_min_ = 2.8°
Absorption correction: multi-scan (*SADABS*; Bruker, 2001)	*h* = −15→15
*T*_min_ = 0.770, *T*_max_ = 1.000	*k* = −17→17
16038 measured reflections	*l* = −17→17

### Refinement

**Table:**

Refinement on *F*^2^	Primary atom site location: structure-invariant direct methods
Least-squares matrix: full	Secondary atom site location: difference Fourier map
*R*[*F*^2^ > 2σ(*F*^2^)] = 0.034	Hydrogen site location: inferred from neighbouring sites
*wR*(*F*^2^) = 0.098	H-atom parameters constrained
*S* = 1.04	*w* = 1/[σ^2^(*F*_o_^2^) + (0.0619*P*)^2^] where *P* = (*F*_o_^2^ + 2*F*_c_^2^)/3
4972 reflections	(Δ/σ)_max_ = 0.001
379 parameters	Δρ_max_ = 0.30 e Å^−^^3^
0 restraints	Δρ_min_ = −0.31 e Å^−^^3^

### Special details

**Table:**

Experimental. IR cm^-1^ (KBr) 3435, 1388; Anal. Calcd for C_13_H_11_ClN_4_O_2_S C, 45.82; H, 2.96; N, 16.44%; Found, C, 45.18; H, 2.92; N, 15.84%.
Geometry. All s.u.'s (except the s.u. in the dihedral angle between two l.s. planes) are estimated using the full covariance matrix. The cell s.u.'s are taken into account individually in the estimation of s.u.'s in distances, angles and torsion angles; correlations between s.u.'s in cell parameters are only used when they are defined by crystal symmetry. An approximate (isotropic) treatment of cell s.u.'s is used for estimating s.u.'s involving l.s. planes.
Refinement. Refinement of *F*^2^ against ALL reflections. The weighted *R*-factor *wR* and goodness of fit *S* are based on *F*^2^, conventional *R*-factors *R* are based on *F*, with *F* set to zero for negative *F*^2^. The threshold expression of *F*^2^ > 2σ(*F*^2^) is used only for calculating *R*-factors(gt) *etc*. and is not relevant to the choice of reflections for refinement. *R*-factors based on *F*^2^ are statistically about twice as large as those based on *F*, and *R*- factors based on ALL data will be even larger.

### Fractional atomic coordinates and isotropic or equivalent isotropic displacement parameters (Å^2^)

**Table:**

	*x*	*y*	*z*	*U*_iso_*/*U*_eq_	
Cl1A	0.20678 (5)	0.52370 (5)	0.40718 (5)	0.06025 (19)	
Cl1B	0.93941 (5)	0.41848 (4)	0.38570 (5)	0.05977 (19)	
S2A	0.28936 (4)	0.22270 (3)	0.58582 (4)	0.03821 (16)	
S2B	0.70463 (4)	0.13503 (3)	0.40663 (4)	0.03622 (15)	
O3A	0.26710 (11)	0.21724 (10)	0.48964 (11)	0.0500 (4)	
O3B	0.70151 (13)	0.19837 (10)	0.47946 (11)	0.0522 (4)	
O4A	0.32556 (11)	0.14347 (9)	0.63449 (13)	0.0562 (5)	
O4B	0.63810 (11)	0.05785 (9)	0.40271 (11)	0.0461 (4)	
N5A	0.51992 (13)	0.58429 (10)	0.64248 (12)	0.0357 (4)	
N5B	0.62508 (13)	0.45541 (11)	0.14780 (12)	0.0369 (4)	
N6A	0.30372 (12)	0.41345 (11)	0.52175 (12)	0.0371 (4)	
N6B	0.79517 (13)	0.30202 (11)	0.33564 (12)	0.0376 (4)	
N7A	0.37871 (13)	0.29776 (10)	0.61344 (13)	0.0439 (5)	
H7A	0.4314	0.2801	0.6480	0.053*	
N7B	0.67403 (13)	0.18711 (11)	0.30975 (12)	0.0394 (4)	
H7B	0.6335	0.1599	0.2693	0.047*	
N8A	0.52770 (13)	0.43954 (10)	0.69189 (12)	0.0360 (4)	
N8B	0.56595 (12)	0.31475 (11)	0.16965 (12)	0.0364 (4)	
C9A	0.17905 (15)	0.26081 (13)	0.63400 (15)	0.0354 (5)	
C9B	0.83185 (15)	0.09728 (13)	0.40267 (14)	0.0354 (5)	
C10A	0.1679 (2)	0.23876 (17)	0.72382 (18)	0.0609 (7)	
H10A	0.2202	0.2094	0.7593	0.073*	
C10B	0.91108 (18)	0.14379 (15)	0.44983 (16)	0.0482 (6)	
H10B	0.8982	0.1949	0.4842	0.058*	
C11A	0.0783 (3)	0.2610 (2)	0.7596 (2)	0.0800 (9)	
H11A	0.0695	0.2463	0.8200	0.096*	
C11B	1.01033 (19)	0.11297 (18)	0.44508 (19)	0.0594 (7)	
H11B	1.0645	0.1433	0.4771	0.071*	
C12A	0.0013 (2)	0.30470 (19)	0.7073 (2)	0.0755 (9)	
H12A	−0.0596	0.3187	0.7321	0.091*	
C12B	1.0294 (2)	0.03867 (19)	0.39389 (19)	0.0615 (7)	
H12B	1.0964	0.0189	0.3909	0.074*	
C13A	0.01382 (19)	0.32755 (18)	0.6197 (2)	0.0641 (7)	
H13A	−0.0383	0.3579	0.5849	0.077*	
C13B	0.9501 (2)	−0.00710 (18)	0.34674 (18)	0.0577 (7)	
H13B	0.9635	−0.0576	0.3117	0.069*	
C14A	0.10334 (16)	0.30612 (15)	0.58193 (17)	0.0458 (6)	
H14A	0.1122	0.3222	0.5219	0.055*	
C14B	0.85071 (18)	0.02174 (14)	0.35123 (16)	0.0458 (6)	
H14B	0.7968	−0.0094	0.3198	0.055*	
C15A	0.37606 (14)	0.38857 (12)	0.58513 (14)	0.0318 (4)	
C15B	0.71004 (15)	0.27352 (12)	0.28880 (13)	0.0321 (5)	
C16A	0.30231 (16)	0.50038 (14)	0.49461 (15)	0.0378 (5)	
C16B	0.82753 (15)	0.38663 (14)	0.32074 (14)	0.0376 (5)	
C17A	0.36691 (15)	0.56812 (13)	0.52574 (14)	0.0356 (5)	
H17A	0.3617	0.6274	0.5041	0.043*	
C17B	0.78186 (16)	0.44845 (13)	0.26077 (14)	0.0367 (5)	
H17B	0.8070	0.5069	0.2538	0.044*	
C18A	0.44159 (15)	0.54000 (12)	0.59313 (13)	0.0298 (4)	
C18B	0.69432 (14)	0.41563 (13)	0.21132 (13)	0.0317 (4)	
C19A	0.44741 (14)	0.45033 (12)	0.62405 (13)	0.0290 (4)	
C19B	0.65665 (14)	0.32852 (12)	0.22385 (13)	0.0309 (4)	
C20A	0.56688 (17)	0.52153 (13)	0.69992 (15)	0.0386 (5)	
H20A	0.6223	0.5356	0.7415	0.046*	
C20B	0.55180 (16)	0.39220 (14)	0.12623 (15)	0.0401 (5)	
H20B	0.4959	0.4029	0.0840	0.048*	
C21A	0.54967 (18)	0.67998 (13)	0.63396 (16)	0.0468 (6)	
H21A	0.5035	0.7092	0.5887	0.070*	
H21B	0.6182	0.6833	0.6159	0.070*	
H21C	0.5467	0.7099	0.6917	0.070*	
C21B	0.62941 (19)	0.54775 (14)	0.11289 (18)	0.0530 (6)	
H21D	0.6901	0.5772	0.1399	0.080*	
H21E	0.5700	0.5808	0.1280	0.080*	
H21F	0.6310	0.5461	0.0477	0.080*	

### Atomic displacement parameters (Å^2^)

**Table:**

	*U*^11^	*U*^22^	*U*^33^	*U*^12^	*U*^13^	*U*^23^
Cl1A	0.0494 (4)	0.0661 (4)	0.0616 (4)	0.0194 (3)	−0.0152 (3)	0.0047 (3)
Cl1B	0.0511 (4)	0.0597 (4)	0.0645 (4)	−0.0155 (3)	−0.0170 (3)	0.0071 (3)
S2A	0.0297 (3)	0.0228 (3)	0.0614 (4)	−0.0001 (2)	−0.0003 (2)	−0.0053 (2)
S2B	0.0421 (3)	0.0276 (3)	0.0390 (3)	−0.0011 (2)	0.0037 (2)	0.0059 (2)
O3A	0.0468 (9)	0.0467 (9)	0.0569 (11)	−0.0055 (7)	0.0075 (8)	−0.0219 (7)
O3B	0.0701 (11)	0.0442 (9)	0.0439 (10)	−0.0004 (8)	0.0137 (8)	−0.0035 (7)
O4A	0.0366 (8)	0.0248 (7)	0.1057 (14)	0.0018 (6)	−0.0022 (9)	0.0105 (8)
O4B	0.0462 (9)	0.0344 (8)	0.0572 (10)	−0.0067 (7)	0.0017 (8)	0.0130 (7)
N5A	0.0416 (10)	0.0235 (8)	0.0423 (11)	−0.0050 (7)	0.0055 (8)	−0.0038 (7)
N5B	0.0379 (10)	0.0332 (9)	0.0396 (10)	0.0005 (8)	0.0031 (8)	0.0124 (8)
N6A	0.0325 (9)	0.0314 (9)	0.0467 (11)	0.0043 (7)	−0.0009 (8)	−0.0028 (8)
N6B	0.0372 (10)	0.0336 (9)	0.0413 (11)	−0.0010 (8)	−0.0012 (8)	0.0063 (7)
N7A	0.0336 (9)	0.0254 (9)	0.0695 (14)	−0.0021 (7)	−0.0125 (9)	0.0058 (8)
N7B	0.0438 (10)	0.0285 (9)	0.0437 (11)	−0.0036 (8)	−0.0083 (8)	0.0071 (7)
N8A	0.0404 (10)	0.0297 (9)	0.0372 (10)	−0.0031 (7)	0.0001 (8)	−0.0019 (7)
N8B	0.0340 (9)	0.0362 (9)	0.0387 (10)	−0.0022 (8)	0.0012 (8)	0.0083 (8)
C9A	0.0366 (11)	0.0258 (10)	0.0434 (13)	−0.0020 (9)	0.0016 (10)	−0.0051 (9)
C9B	0.0400 (11)	0.0297 (10)	0.0356 (12)	−0.0018 (9)	−0.0016 (9)	0.0104 (9)
C10A	0.0735 (18)	0.0581 (15)	0.0509 (17)	0.0107 (14)	0.0045 (14)	0.0047 (12)
C10B	0.0552 (15)	0.0413 (12)	0.0451 (14)	−0.0063 (11)	−0.0117 (11)	0.0010 (10)
C11A	0.112 (3)	0.075 (2)	0.059 (2)	0.0138 (19)	0.0413 (19)	0.0038 (15)
C11B	0.0455 (14)	0.0673 (17)	0.0621 (18)	−0.0127 (13)	−0.0139 (12)	0.0145 (14)
C12A	0.070 (2)	0.0649 (18)	0.098 (3)	0.0096 (15)	0.0439 (19)	−0.0142 (17)
C12B	0.0413 (14)	0.0714 (18)	0.072 (2)	0.0066 (13)	0.0078 (13)	0.0200 (15)
C13A	0.0477 (15)	0.0657 (17)	0.080 (2)	0.0208 (13)	0.0115 (14)	−0.0072 (15)
C13B	0.0556 (16)	0.0572 (15)	0.0608 (17)	0.0078 (13)	0.0083 (13)	−0.0008 (13)
C14A	0.0399 (12)	0.0465 (13)	0.0508 (15)	0.0105 (10)	0.0024 (11)	−0.0005 (11)
C14B	0.0480 (14)	0.0406 (12)	0.0478 (14)	−0.0033 (10)	−0.0011 (11)	−0.0016 (10)
C15A	0.0319 (10)	0.0243 (9)	0.0393 (12)	0.0022 (8)	0.0044 (9)	−0.0036 (8)
C15B	0.0335 (11)	0.0271 (10)	0.0362 (12)	0.0017 (9)	0.0061 (9)	0.0040 (8)
C16A	0.0350 (11)	0.0386 (12)	0.0394 (13)	0.0117 (10)	0.0013 (10)	−0.0018 (9)
C16B	0.0346 (11)	0.0399 (12)	0.0381 (13)	−0.0043 (9)	0.0028 (9)	0.0002 (9)
C17A	0.0401 (11)	0.0287 (10)	0.0394 (12)	0.0078 (9)	0.0106 (10)	0.0025 (9)
C17B	0.0390 (11)	0.0317 (10)	0.0400 (13)	−0.0066 (9)	0.0065 (10)	0.0053 (9)
C18A	0.0345 (11)	0.0264 (10)	0.0301 (11)	0.0021 (8)	0.0117 (9)	−0.0037 (8)
C18B	0.0344 (10)	0.0299 (10)	0.0318 (11)	0.0005 (9)	0.0092 (9)	0.0065 (8)
C19A	0.0310 (10)	0.0269 (10)	0.0297 (11)	0.0007 (8)	0.0063 (9)	−0.0019 (8)
C19B	0.0306 (10)	0.0298 (10)	0.0330 (12)	0.0012 (8)	0.0066 (9)	0.0024 (8)
C20A	0.0412 (12)	0.0351 (11)	0.0379 (13)	−0.0038 (9)	−0.0044 (10)	−0.0052 (9)
C20B	0.0350 (11)	0.0429 (12)	0.0412 (13)	−0.0033 (10)	−0.0034 (10)	0.0116 (10)
C21A	0.0570 (14)	0.0276 (11)	0.0568 (15)	−0.0101 (10)	0.0108 (12)	−0.0030 (10)
C21B	0.0605 (15)	0.0373 (12)	0.0606 (16)	−0.0016 (11)	0.0013 (13)	0.0230 (11)

### Geometric parameters (Å, º)

**Table:**

Cl1A—C16A	1.746 (2)	C10B—C11B	1.385 (3)
Cl1B—C16B	1.741 (2)	C10B—H10B	0.9300
S2A—O3A	1.4198 (17)	C11A—C12A	1.373 (4)
S2A—O4A	1.4273 (15)	C11A—H11A	0.9300
S2A—N7A	1.6347 (17)	C11B—C12B	1.363 (4)
S2A—C9A	1.757 (2)	C11B—H11B	0.9300
S2B—O3B	1.4230 (16)	C12A—C13A	1.354 (4)
S2B—O4B	1.4305 (14)	C12A—H12A	0.9300
S2B—N7B	1.6352 (17)	C12B—C13B	1.373 (4)
S2B—C9B	1.763 (2)	C12B—H12B	0.9300
N5A—C20A	1.361 (3)	C13A—C14A	1.379 (3)
N5A—C18A	1.368 (2)	C13A—H13A	0.9300
N5A—C21A	1.470 (2)	C13B—C14B	1.377 (3)
N5B—C20B	1.354 (3)	C13B—H13B	0.9300
N5B—C18B	1.374 (2)	C14A—H14A	0.9300
N5B—C21B	1.456 (2)	C14B—H14B	0.9300
N6A—C15A	1.320 (2)	C15A—C19A	1.390 (3)
N6A—C16A	1.341 (3)	C15B—C19B	1.392 (3)
N6B—C15B	1.326 (3)	C16A—C17A	1.361 (3)
N6B—C16B	1.340 (2)	C16B—C17B	1.367 (3)
N7A—C15A	1.400 (2)	C17A—C18A	1.392 (3)
N7A—H7A	0.8600	C17A—H17A	0.9300
N7B—C15B	1.401 (2)	C17B—C18B	1.389 (3)
N7B—H7B	0.8600	C17B—H17B	0.9300
N8A—C20A	1.313 (2)	C18A—C19A	1.396 (3)
N8A—C19A	1.392 (3)	C18B—C19B	1.393 (3)
N8B—C20B	1.312 (2)	C20A—H20A	0.9300
N8B—C19B	1.385 (2)	C20B—H20B	0.9300
C9A—C14A	1.371 (3)	C21A—H21A	0.9600
C9A—C10A	1.380 (3)	C21A—H21B	0.9600
C9B—C10B	1.378 (3)	C21A—H21C	0.9600
C9B—C14B	1.380 (3)	C21B—H21D	0.9600
C10A—C11A	1.369 (4)	C21B—H21E	0.9600
C10A—H10A	0.9300	C21B—H21F	0.9600
			
O3A—S2A—O4A	118.82 (10)	C12B—C13B—H13B	120.0
O3A—S2A—N7A	111.37 (10)	C14B—C13B—H13B	120.0
O4A—S2A—N7A	103.28 (9)	C9A—C14A—C13A	119.1 (2)
O3A—S2A—C9A	108.41 (10)	C9A—C14A—H14A	120.5
O4A—S2A—C9A	107.92 (10)	C13A—C14A—H14A	120.5
N7A—S2A—C9A	106.31 (9)	C9B—C14B—C13B	119.4 (2)
O3B—S2B—O4B	119.51 (9)	C9B—C14B—H14B	120.3
O3B—S2B—N7B	109.03 (9)	C13B—C14B—H14B	120.3
O4B—S2B—N7B	103.68 (9)	N6A—C15A—C19A	121.52 (17)
O3B—S2B—C9B	108.80 (10)	N6A—C15A—N7A	118.16 (17)
O4B—S2B—C9B	108.84 (9)	C19A—C15A—N7A	120.32 (18)
N7B—S2B—C9B	106.16 (9)	N6B—C15B—C19B	121.33 (17)
C20A—N5A—C18A	106.34 (15)	N6B—C15B—N7B	117.25 (17)
C20A—N5A—C21A	126.50 (18)	C19B—C15B—N7B	121.38 (18)
C18A—N5A—C21A	127.14 (18)	N6A—C16A—C17A	127.47 (19)
C20B—N5B—C18B	106.11 (15)	N6A—C16A—Cl1A	113.37 (16)
C20B—N5B—C21B	127.47 (19)	C17A—C16A—Cl1A	119.14 (16)
C18B—N5B—C21B	126.40 (18)	N6B—C16B—C17B	126.69 (19)
C15A—N6A—C16A	117.71 (17)	N6B—C16B—Cl1B	115.09 (16)
C15B—N6B—C16B	118.25 (18)	C17B—C16B—Cl1B	118.22 (15)
C15A—N7A—S2A	125.03 (15)	C16A—C17A—C18A	113.45 (18)
C15A—N7A—H7A	117.5	C16A—C17A—H17A	123.3
S2A—N7A—H7A	117.5	C18A—C17A—H17A	123.3
C15B—N7B—S2B	123.79 (15)	C16B—C17B—C18B	113.53 (18)
C15B—N7B—H7B	118.1	C16B—C17B—H17B	123.2
S2B—N7B—H7B	118.1	C18B—C17B—H17B	123.2
C20A—N8A—C19A	102.94 (16)	N5A—C18A—C17A	133.00 (17)
C20B—N8B—C19B	103.25 (16)	N5A—C18A—C19A	105.18 (17)
C14A—C9A—C10A	121.0 (2)	C17A—C18A—C19A	121.82 (18)
C14A—C9A—S2A	120.72 (17)	N5B—C18B—C17B	132.22 (17)
C10A—C9A—S2A	118.14 (18)	N5B—C18B—C19B	105.20 (17)
C10B—C9B—C14B	120.8 (2)	C17B—C18B—C19B	122.51 (18)
C10B—C9B—S2B	119.93 (17)	N8A—C19A—C15A	131.09 (17)
C14B—C9B—S2B	119.24 (16)	N8A—C19A—C18A	110.89 (17)
C11A—C10A—C9A	118.7 (3)	C15A—C19A—C18A	118.02 (18)
C11A—C10A—H10A	120.7	N8B—C19B—C18B	110.79 (17)
C9A—C10A—H10A	120.7	N8B—C19B—C15B	131.45 (17)
C9B—C10B—C11B	118.7 (2)	C18B—C19B—C15B	117.66 (18)
C9B—C10B—H10B	120.6	N8A—C20A—N5A	114.65 (19)
C11B—C10B—H10B	120.6	N8A—C20A—H20A	122.7
C10A—C11A—C12A	120.6 (3)	N5A—C20A—H20A	122.7
C10A—C11A—H11A	119.7	N8B—C20B—N5B	114.65 (18)
C12A—C11A—H11A	119.7	N8B—C20B—H20B	122.7
C12B—C11B—C10B	120.6 (2)	N5B—C20B—H20B	122.7
C12B—C11B—H11B	119.7	N5A—C21A—H21A	109.5
C10B—C11B—H11B	119.7	N5A—C21A—H21B	109.5
C13A—C12A—C11A	120.2 (2)	H21A—C21A—H21B	109.5
C13A—C12A—H12A	119.9	N5A—C21A—H21C	109.5
C11A—C12A—H12A	119.9	H21A—C21A—H21C	109.5
C11B—C12B—C13B	120.3 (2)	H21B—C21A—H21C	109.5
C11B—C12B—H12B	119.8	N5B—C21B—H21D	109.5
C13B—C12B—H12B	119.8	N5B—C21B—H21E	109.5
C12A—C13A—C14A	120.4 (3)	H21D—C21B—H21E	109.5
C12A—C13A—H13A	119.8	N5B—C21B—H21F	109.5
C14A—C13A—H13A	119.8	H21D—C21B—H21F	109.5
C12B—C13B—C14B	120.0 (2)	H21E—C21B—H21F	109.5
			
O3A—S2A—N7A—C15A	54.0 (2)	C15B—N6B—C16B—C17B	−0.4 (3)
O4A—S2A—N7A—C15A	−177.38 (17)	C15B—N6B—C16B—Cl1B	179.63 (15)
C9A—S2A—N7A—C15A	−63.9 (2)	N6A—C16A—C17A—C18A	0.2 (3)
O3B—S2B—N7B—C15B	42.58 (18)	Cl1A—C16A—C17A—C18A	−177.92 (14)
O4B—S2B—N7B—C15B	170.92 (16)	N6B—C16B—C17B—C18B	−1.1 (3)
C9B—S2B—N7B—C15B	−74.47 (18)	Cl1B—C16B—C17B—C18B	178.91 (15)
O3A—S2A—C9A—C14A	−20.6 (2)	C20A—N5A—C18A—C17A	179.0 (2)
O4A—S2A—C9A—C14A	−150.49 (17)	C21A—N5A—C18A—C17A	−2.4 (3)
N7A—S2A—C9A—C14A	99.25 (18)	C20A—N5A—C18A—C19A	−0.8 (2)
O3A—S2A—C9A—C10A	155.31 (17)	C21A—N5A—C18A—C19A	177.74 (17)
O4A—S2A—C9A—C10A	25.4 (2)	C16A—C17A—C18A—N5A	−179.73 (19)
N7A—S2A—C9A—C10A	−84.85 (19)	C16A—C17A—C18A—C19A	0.1 (3)
O3B—S2B—C9B—C10B	−14.6 (2)	C20B—N5B—C18B—C17B	−176.7 (2)
O4B—S2B—C9B—C10B	−146.40 (17)	C21B—N5B—C18B—C17B	2.1 (3)
N7B—S2B—C9B—C10B	102.56 (18)	C20B—N5B—C18B—C19B	0.0 (2)
O3B—S2B—C9B—C14B	166.09 (16)	C21B—N5B—C18B—C19B	178.77 (19)
O4B—S2B—C9B—C14B	34.3 (2)	C16B—C17B—C18B—N5B	177.4 (2)
N7B—S2B—C9B—C14B	−76.70 (18)	C16B—C17B—C18B—C19B	1.2 (3)
C14A—C9A—C10A—C11A	1.7 (4)	C20A—N8A—C19A—C15A	−179.5 (2)
S2A—C9A—C10A—C11A	−174.2 (2)	C20A—N8A—C19A—C18A	0.2 (2)
C14B—C9B—C10B—C11B	−0.4 (3)	N6A—C15A—C19A—N8A	178.71 (19)
S2B—C9B—C10B—C11B	−179.61 (18)	N7A—C15A—C19A—N8A	−0.5 (3)
C9A—C10A—C11A—C12A	−0.3 (4)	N6A—C15A—C19A—C18A	−1.0 (3)
C9B—C10B—C11B—C12B	0.7 (4)	N7A—C15A—C19A—C18A	179.78 (17)
C10A—C11A—C12A—C13A	−0.9 (5)	N5A—C18A—C19A—N8A	0.4 (2)
C10B—C11B—C12B—C13B	−0.5 (4)	C17A—C18A—C19A—N8A	−179.47 (17)
C11A—C12A—C13A—C14A	0.8 (4)	N5A—C18A—C19A—C15A	−179.87 (16)
C11B—C12B—C13B—C14B	−0.2 (4)	C17A—C18A—C19A—C15A	0.3 (3)
C10A—C9A—C14A—C13A	−1.7 (3)	C20B—N8B—C19B—C18B	0.4 (2)
S2A—C9A—C14A—C13A	174.03 (18)	C20B—N8B—C19B—C15B	176.6 (2)
C12A—C13A—C14A—C9A	0.5 (4)	N5B—C18B—C19B—N8B	−0.3 (2)
C10B—C9B—C14B—C13B	−0.3 (3)	C17B—C18B—C19B—N8B	176.86 (18)
S2B—C9B—C14B—C13B	178.94 (18)	N5B—C18B—C19B—C15B	−177.09 (17)
C12B—C13B—C14B—C9B	0.6 (4)	C17B—C18B—C19B—C15B	0.0 (3)
C16A—N6A—C15A—C19A	1.2 (3)	N6B—C15B—C19B—N8B	−177.66 (19)
C16A—N6A—C15A—N7A	−179.50 (18)	N7B—C15B—C19B—N8B	0.0 (3)
S2A—N7A—C15A—N6A	−9.7 (3)	N6B—C15B—C19B—C18B	−1.6 (3)
S2A—N7A—C15A—C19A	169.53 (15)	N7B—C15B—C19B—C18B	176.01 (17)
C16B—N6B—C15B—C19B	1.8 (3)	C19A—N8A—C20A—N5A	−0.8 (2)
C16B—N6B—C15B—N7B	−175.93 (17)	C18A—N5A—C20A—N8A	1.1 (2)
S2B—N7B—C15B—N6B	21.4 (2)	C21A—N5A—C20A—N8A	−177.51 (18)
S2B—N7B—C15B—C19B	−156.28 (15)	C19B—N8B—C20B—N5B	−0.4 (2)
C15A—N6A—C16A—C17A	−0.9 (3)	C18B—N5B—C20B—N8B	0.2 (2)
C15A—N6A—C16A—Cl1A	177.34 (15)	C21B—N5B—C20B—N8B	−178.51 (19)

### Hydrogen-bond geometry (Å, º)

**Table:**

*D*—H···*A*	*D*—H	H···*A*	*D*···*A*	*D*—H···*A*
N7*A*—H7*A*···N8*B*^i^	0.86	2.25	3.014 (2)	148
N7*B*—H7*B*···N8*A*^ii^	0.86	2.25	3.092 (2)	165
C13*A*—H13*A*···Cl1*A*^iii^	0.93	2.82	3.620 (3)	144
C20*A*—H20*A*···O4*A*^iv^	0.93	2.47	3.243 (3)	141

Symmetry codes: (i) *x*, −*y*+1/2, *z*+1/2; (ii) *x*, −*y*+1/2, *z*−1/2; (iii) −*x*, −*y*+1, −*z*+1; (iv) −*x*+1, *y*+1/2, −*z*+3/2.
